# The complete mitogenome of the pond wolf spider *Pardosa pseudoannulata*, with phylogenetic implications for the Lycosidae

**DOI:** 10.1080/23802359.2024.2337791

**Published:** 2024-04-10

**Authors:** Yufa Luo, Ying Cheng, Lijuan Liu, Dan Fu

**Affiliations:** School of Life and Environmental Sciences, Key Laboratory of Wetland Biodiversity of the Jianhu Basin of Shaoxing, Shaoxing University, Shaoxing, Zhejiang, China

**Keywords:** Araneae, *Pardosa pseudoannulata*, phylogeny, Illumina sequencing

## Abstract

The pond wolf spider *Pardosa pseudoannulata* Bösenberg & Strand, 1906 (Araneae: Lycosidae) is an important predator of agricultural pests in southern, eastern and southeastern Asia. Here, we report the complete mitogenome of this spider reconstructed from Illumina sequencing data. The circular mitogenome length is 14,533 bp with the nucleotide composition A (33.3%), C (8.2%), G (15.2%), and T (43.3%). The *P. pseudoannulata* mitogenome comprises 13 protein-coding genes, 22 transfer RNA genes, two ribosomal RNA genes, and a control region. Phylogenetic analyses of Lycosidae mitogenomes supported the monophyly of the subfamily Pardosinae and the two genera *Pardosa* and *Alopecosa*, and indicated the polyphyly of the subfamily Lycosinae and the paraphyly of its type genus *Lycosa*. In this study, *P. pseudoannulata* is the closest relative to *P. pusiola*. These results provide useful genetic information for future studies on the diversity, phylogeny, and evolution for wolf spiders.

## 1. Introduction

Globally, spiders are composed of 135 families, 4,376 genera, and 51,993 species (World Spider Catalog 2024). They are important predators for the insect pests of agriculture, forestry, fruit trees, and other economically important plants (Yang et al. 2018; Li et al. 2021). The wolf spider familar, Lycosidae is the fifth largest group in the order Araneae with 133 genera, and 2,478 species (World Spider Catalog 2024). *Pardosa* is the largest genus in the family with 532 species, and the spiders of this genus distribute worldwide (World Spider Catalog 2024). The pond wolf spider *P. pseudoannulata* Bösenberg & Strand, 1906 is an Asian species from South, Southeast and East Asia (Song et al. 1999; World Spider Catalog 2024). It preys on many species of insect pests that are of agricultural importance and, as a consequence, this spider has been looked as a possible biological control agent (Li et al. 2017). The increasing interest in the potential of spider venom as a tool for pest control have prompted a detailed study on this spider (Huang et al. 2018). In this study, we sequenced a *P. pseudoannulata* mitogenome, and analyzed the phylogenetic relationships of the major subfamilies and genera within Lycosidae using mitogenomes. These results will be useful in the future studies for phylogenetics, population genetics, and biogeography within Araneae.

## 2. Materials and methods

### 2.1. Sample collection

**Figure 1.**
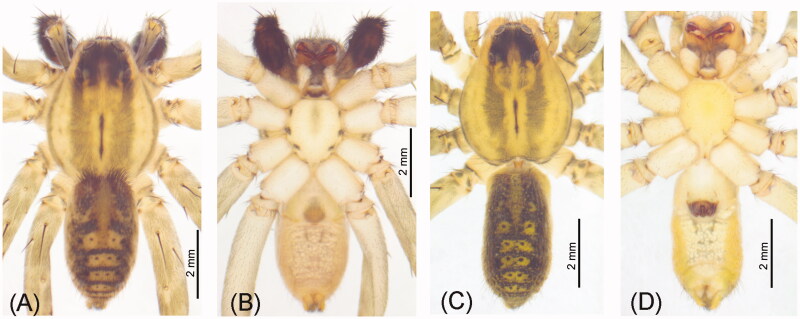
The reference images of *P. pseudoannulata* Bösenberg & Strand, 1906 in this work, collected by Ying Cheng in Yuecheng District (N29°58′54″, E120°34′03″), Shaoxing, China. Photos taken by Ying Cheng at the Zoological Museum of Shaoxing University, Shaoxing, China. (A) Male: dorsal view; (B) male: ventral view; (C) female: dorsal view; (D) female: ventral view.

The adult specimens of *P. pseudoannulata* (Figure 1) were collected from the rice fields (N29°58′54″, E120°34′03″) in Yuecheng District, Shaoxing City, Zhejiang Province of China in mid-October 2022. After morphological identification, the specimens (voucher number: SXU-SHS-2022LCG01, contact person: Yufa Luo, lyf223@126.com) were stored in absolute ethanol, and placed in a freezer at −20 °C in the Zoological Museum (Arachnological collection) of Shaoxing University, Shaoxing, China.

### 2.2. DNA extraction and sequencing

Total genomic DNA was extracted from the cephalothorax and legs of the male sample using the modified cetyltrimethyl ammonium bromide (CTAB) method (Shahjahan et al. 1995). The Illumina Novoseq 6000 sequencing platform was used for sequencing, and the Illumina PE150 library was constructed at Personalbio Biotechnology Company (Shanghai, China).

### 2.3. Genome assembly, annotation and phylogenetic analysis

The complete mitogenome of *P. pseudoannulata* was assembled from next-generation sequence data using Geneious Prime v2023.1.2 according to the reference mitogenome of *P. laura* (GenBank accession number: KM272948). Gene annotation of the assembled mitogenome was performed using MITOS website (http://mitos.bioinf.uni-leipzig.de/index.py, Bernt et al. 2013). The 13 protein-coding genes (PCGs) were annotated and reviewed by searching the NCBI non-redundant protein sequence database using BLAST (Altschul et al. 1997). We determined the 22 tRNA gene sequences using the MITOS website and ARWEN v1.2 (Laslett and Canbäck 2008). The circular mitogenome map was drawn by Organellar Genome DRAW (OGDRAW) v1.3.1 (https://chlorobox.mpimp-golm.mpg.de/OGDraw.html; Greiner et al. 2019).

In order to understand the phylogenetic status of *P. pseudoannulata* and the phylogeny of major subfamilies and genera within Lycosidae, we downloaded the 16 lycosid mitogenomes from the NCBI database. The mitogenomes of *Pisaura bicornis* (GenBank accession number: MN296112) and *Dolomedes angustivirgatus* (GenBank accession number: NC031355) of Pisauridae were selected as outgroups. Maximum-likelihood (ML) method in W-IQ-TREE 2.0 (Hoang et al. 2018, 1000 ultrafast bootstrap) was used to reconstruct phylogenetic tree using the 13 PCGs. Sequences were aligned using MAFFT 7 (http://mafft.cbrc.jp/alignment/server/) with the L-INS-i method. To detect and exclude ambiguously aligned regions, alignments of the 13 PCGs were processed with the program trimAl v1.3 (Capella-Gutiérrez et al. 2009). We inferred ML topologies based on the partitioning strategy. For each gene partition, the best-fitting substitution model was estimated simultaneously using the greedy algorithm in ModelFinder (Kalyaanamoorthy et al. 2017). We used the Bayesian information criterion and the FreeRate heterogeneity to select the best-fitting substitution model for each gene partition (TIM + F + I + G4 for ATP6, CYTB and ND2; TN + F + G4 for ATP8; GTR + F + R3 for COX1; TIM + F + R3 for COX2; TIM3 + F + R3 for COX3 and ND1; TIM3 + F + I + G4 for ND3, ND4, ND4L and ND5; and TVM + F + I + G4 for ND6). The perturbation strength (*p*) and the number of iterations since the last best tree found (*c*) were set to 0.3 and 1000, respectively.

## 3. Results

### 3.1. Mitogenomic characteristics

**Figure 2.**
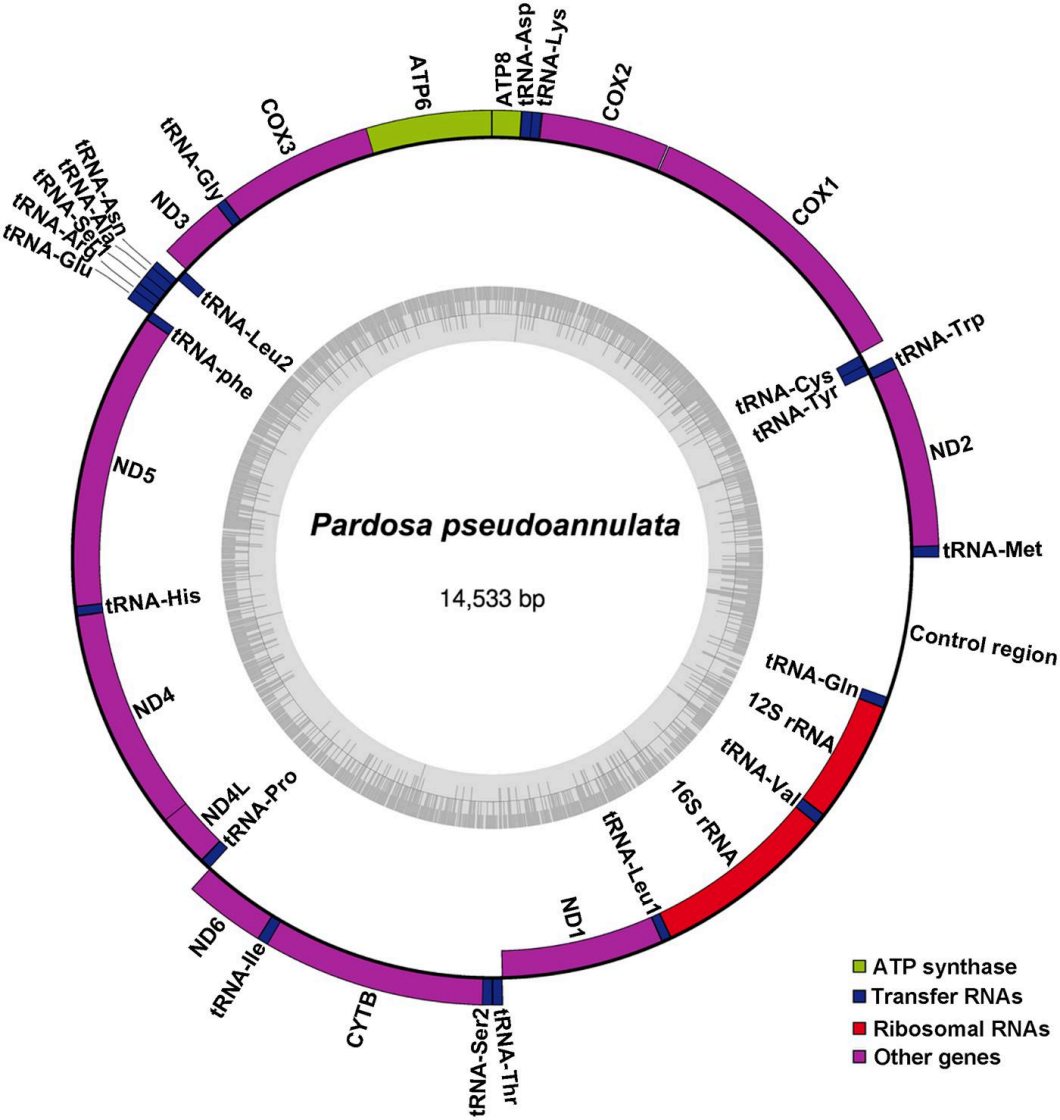
The circular map of the *P. pseudoannulata* mitogenome. The inner ring indicates the GC content.

The complete mitogenome of *P. pseudoannulata* (GenBank accession number: OR888932) is 14,533 bp in length, and shows a high nucleotide bias with 76.6% of A + T and 23.4% of G + C (33.3% A; 43.3% T; 15.2% G; and 8.2% C). The mitogenome consists of 37 genes (13 PCGs, 22 transfer RNA genes, and two ribosomal RNA genes) and a control region (Figure 2 and Supplementary Figure S1). Among the 37 genes, 22 are encoded on the major strand (J-strand) while the others are encoded on the minor strand (N-strand). Most of PCGs start with the ATA (COX1, COX2, ATP6, ND3, ND4, and ND4L) or TTG (ND2, COX3, and ND6); ND5 and ND1 start with the ATT; ATP8 and CYTB start with the ATG. Seven PCGs are terminated with the TAA (COX1, COX2, ATP8, ATP6, COX3, ND3, and CYTB), and six end from an incomplete stop codon (ND2, ND5, ND4, ND4L, ND6, and ND1). The gene order of the *P. pseudoannulata* mitogenome is identical to that of other wolf spiders (Ye et al. 2022).

### 3.2. Phylogenetic indications

**Figure 3.**
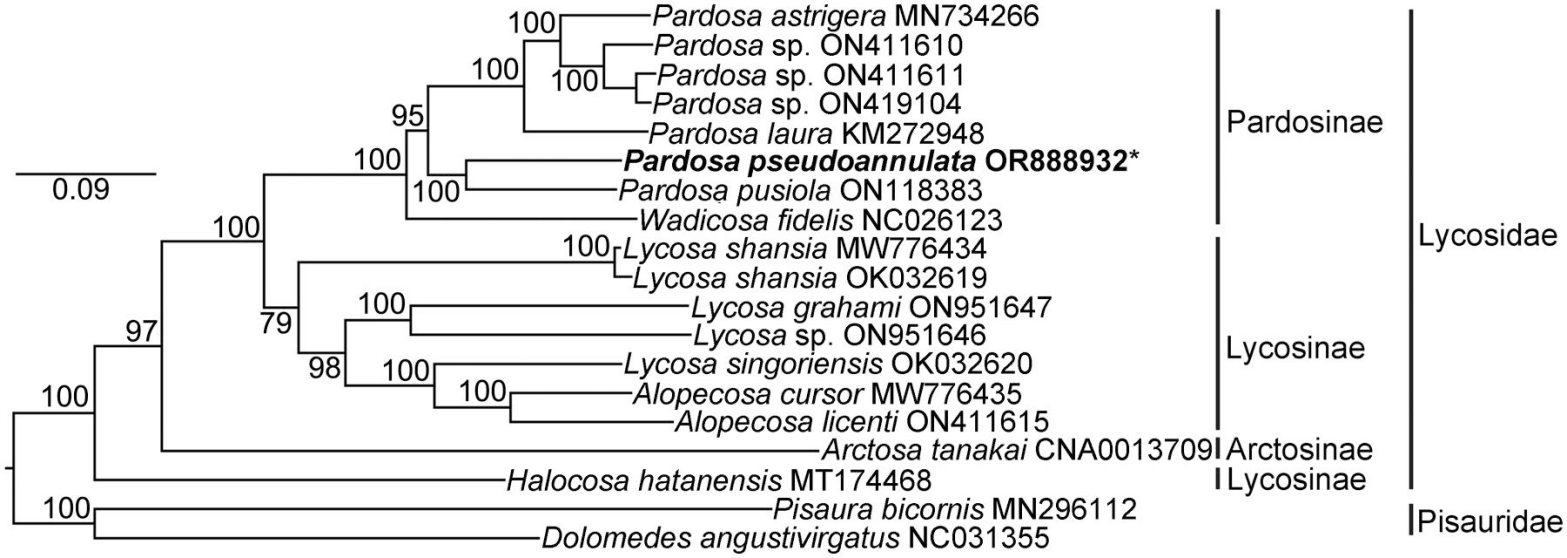
The ML phylogenetic tree reconstructed using the 13 PCGs (the sequenced *P. pseudoannulata* mitogenome by this study marked with asterisk). *Pisaura bicornis* and *Dolomedes angustivirgatus* (Pisauridae) were used as outgroups. The scale-bar indicates 0.09 substitutions per site. The numbers to the left of each node denote the ultrafast bootstrap value for ML analysis. The following sequences were used: ON411610 (Li et al. 2022), ON411611 (Li et al. 2022), ON419104 (Li et al. 2022), MW776434 (Li et al. 2022), and OK032619 (Ye et al. 2022).

The phylogeny of Lycosidae including 17 species of six genera was reconstructed using the 13 PCGs (Figure 3). Our result indicates that *P. pseudoannulata* and *Wadicosa fidelis* belong to the subfamily Pardosinae, and supported the monophyly of Pardosinae. Both *Pardosa* and *Alopecosa* are monophyletic. The genus *Lycosa* is paraphyletic. *Alopecosa* and *Lycosa* species clustered together, and Lycosinae is a polyphyletic group. The genus *Halocosa* may not belong to the subfamily Lycosinae.

## 4. Discussion and conclusions

Lycosid spiders constitute an extensive and diverse branch of the order Araneae. Whereas the mitogenomes of only 16 species of wolf spiders have been determined. Our results showed that the mitogenomic structure of the pond wolf spider *P. pseudoannulata* was similar to that of other lycosid species (Ding et al. 2022; Ye et al. 2023; Yi et al. 2023). The reconstructed phylogenetic tree illuminated the positions of *P. pseudoannulata* and *W. fidelis* in subfamily Pardosinae of Lycosidae, and the phylogenetic relationships between 17 species of the six lycosid genera, and indicated the subfamily Lycosinae and the genus *Lycosa* were both non-monophyletic. Azarkina and Trilikauskas (2019) assigned *Halocosa* to the subfamily Lycosinae based on the latero-median origin of the embolus that is situated in a shallow and wide depression. However, our molecular phylogeny indicated that *Halocosa hatanensis* may not be a species of Lycosinae. The results of this study will contribute to future studies on phylogenetics, population genetics, and biogeography within the family Lycosidae.

## Supplementary Material

Supplemental Material

## Data Availability

The genome sequence data supporting the findings of this study are openly available in the GenBank of NCBI at https://www.ncbi.nlm.nih.gov/genbank/ under the accession OR888932. The associated Bio-Project, SRA, and Bio-sample numbers are PRJNA987849, SRR25033381, and SAMN35994079, respectively.
