# Supplementary figures and images for: The complete mitogenome of the pond wolf spider Pardosa pseudoannulata, with phylogenetic implications for the Lycosidae
Source: Mitochondrial DNA B Resour. 2024 Apr 10;9(4):475–8. doi: 10.1080/23802359.2024.2337791 (PMC11011225; doi:10.1080/23802359.2024.2337791)

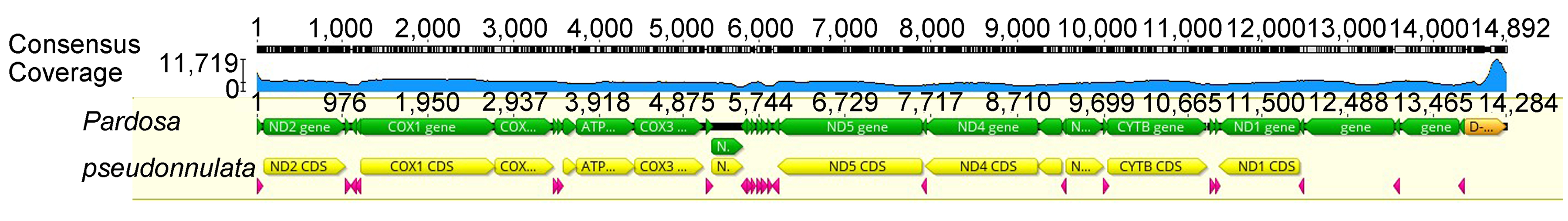

Supplement: Supplemental Material [file TMDN_A_2337791_SM3655.tif]
